# The ConsuMEER study: a randomised trial towards the effectiveness of protein-rich ready-made meals and protein-rich dairy products in increasing protein intake of community-dwelling older adults after switching from self-prepared meals towards ready-made meals

**DOI:** 10.1017/jns.2019.27

**Published:** 2019-09-05

**Authors:** Jos W. Borkent, Janne Beelen, Joost O. Linschooten, Annet J. C. Roodenburg, Marian A. E. de van der Schueren

**Affiliations:** 1 Department of Nutrition and Health, HAN University of Applied Sciences, Faculty of Health and Social Studies, Nijmegen, the Netherlands; 2 Department of Food Science & Technology, HAS University of Applied Sciences, ‘s-Hertogenbosch, The Netherlands

**Keywords:** Undernutrition, Ready-made meals, Community-dwelling older adults, Protein-rich products, Nutritional status, Meals-on-wheels, Home-delivered meals, BW, body weight, DNFCSOA, Dutch National Food Consumption Survey Older Adults, LAPAQ, Longitudinal Ageing Study Amsterdam Physical Activity Questionnaire, LASA, Longitudinal Ageing Study Amsterdam, MMSE, Mini Mental State Examination, SCREEN II, Seniors in the Community: Risk evaluation for eating and nutrition, version II, SNAQ65+, Short Nutritional Assessment Questionnaire 65+, T1, 2 weeks after start of intervention, T2, 4 weeks after start of intervention

## Abstract

The risk of undernutrition in older community-dwelling adults increases when they are no longer able to shop or cook themselves. Home-delivered products could then possibly prevent them from becoming undernourished. This single-blind randomised trial tested the effectiveness of home-delivered protein-rich ready-made meals and dairy products in reaching the recommended intake of 1·2 g protein/kg body weight (BW) per d and ≥25 g of protein per meal. Community-dwelling older adults (*n* 98; mean age 80·4 (sd 6·8) years) switched from self-prepared to home-delivered hot meals and dairy products for 28 d. The intervention group received ready-made meals and dairy products high in protein; the control group received products lower in protein. Dietary intake was measured at baseline, after 2 weeks (T1), and after 4 weeks (T2). Multilevel analyses (providing one combined outcome for T1 and T2) and logistic regressions were performed. Average baseline protein intake was 1·09 (se 0·05) g protein/kg BW per d in the intervention group and 0·99 (se 0·05) g protein/kg BW per d in the control group. During the trial, protein intake of the intervention group was 1·12 (se 0·05) g protein/kg BW per d compared with 0·87 (se 0·03) g protein/kg BW per d in the control group (between-group differences *P* < 0·05). More participants of the intervention group reached the threshold of ≥25 g protein at dinner compared with the control group (intervention T1: 84·8 %, T2: 88·4 % *v.* control T1: 42·9 %, T2: 40·5 %; *P* < 0·05), but not at breakfast and lunch. Our findings suggest that switching from self-prepared meals to ready-made meals carries the risk of a decreasing protein intake, unless extra attention is given to protein-rich choices.

The risk of undernutrition among community-dwelling older adults in developed countries is shown to be as high as 24 %, with an even higher risk and prevalence among frail older adults^(1)^. Protein–energy wasting is the main cause of undernutrition among older adults^(2–4)^ and is induced by a reduced energy and protein intake^(4)^.

The average protein intake of community-dwelling healthy older adults in the Netherlands is 0·9 g protein/kg body weight (BW) per d^(5)^. Although this is above the recommended daily intake of 0·8 g protein/kg BW per d, approximately 20 % of all older adults do not reach this level^(6)^. Moreover, international groups of experts argue that the current recommendations do not fulfil the needs of older adults; an intake of 1·0–1·2 g protein/kg BW per d is recommended for healthy older adults and in the case of illness 1·2–1·5 g protein/kg BW per d or even higher is advised^(7,8)^. Furthermore, not only the total intake per d, but also the distribution of protein intake is said to be important: an intake of at least 25–30 g of protein per meal is thought to be optimal in stimulating muscle protein synthesis^(7,9)^.

There are different reasons why community-dwelling older adults do not meet the recommended protein intake. Well-known factors affecting nutritional intake are physical, psychological, social and/or medical problems^(10,11)^. Impaired mobility, for example, affects older adults' possibilities to do groceries and prepare meals^(12)^. This could lead to a situation where community-dwelling older adults will have to rely on home-delivered ready-made meals^(13)^.

The impact of switching from self-made meals to ready-made meals in terms of protein intake is unclear. Most previous research on the topic of ready-made meals and protein intake is cross-sectional and therefore does not provide evidence about causality of switching from self-made meals to ready-made meals^(14)^. Intervention studies on this topic are scarce and not generalisable as most studies were performed in the USA, involving economically disadvantaged populations, where ready-made meals were provided as a part of a welfare programme^(15)^.

Sharkey & Haines^(16)^ found that a large group of older adults who are regular customers of meal-delivery services suffer from multiple health limitations. Herewith, a recommended intake of over 1·2 g protein/kg BW per d would be optimal for this vulnerable population^(7)^. Previous Dutch intervention studies in older adults provided evidence that ready-made meals and desserts, enriched with extra protein, could increase protein intake towards 1·2 g protein/kg BW per d^(17,18)^. However, these studies cannot be generalised to a community setting as one study was performed in a rehabilitation setting^(18)^ and the enriched products of both studies are mostly not commercially available.

While a low intake or a low protein content of ready-made meals could be a risk for a (too) low protein intake, meals high in protein could contribute to optimising the protein intake especially when combined with a dessert rich in protein^(6)^. Therefore, the aim of the present study is to test the effectiveness of commercially available protein-rich ready-made meals and protein-rich dairy products compared with standard ready-made meals and dairy products lower in protein in reaching the protein goal of 1·2 g protein/kg BW per d for older adults.

## Methods

### Study design and participants

This study was performed as a single-blind, randomised trial with a parallel design. The aim of the study was to test the effectiveness of home-delivered protein-rich ready-made meals and dairy products in reaching the protein goals for older adults, compared with standard ready-made meals and dairy products lower in protein.

Participants were recruited via the database of a meal delivery service (http://www.maaltijdservice.nl) and via advertisements in local newspapers in February and March 2017. All participants had to meet the following inclusion criteria: aged 65 years or over; living at home; being able to eat independently; having a microwave to heat meals; being able to understand, read and speak Dutch. Exclusion criteria were: following a diet with protein restriction or a vegetarian diet; allergies or intolerances prohibiting the use of dairy products; only using texture-modified foods or a liquid diet; diagnosed with renal insufficiency; suffering from a terminal illness; Mini Mental State Examination (MMSE) score <24 (exception: within couples, one participant with a score below 24 was allowed if the partner scored at least 24 points and helped with the food diary).

### Randomisation and blinding

A randomisation scheme was generated by using a website (http://www.randomization.com). Stratified randomisation was performed by group (male, female or couple) and in permuted blocks of eight for male and female and four for couples. Participants were allocated by a 1:1 ratio to the intervention or control group. A researcher who was not aware of the allocation sequence performed the inclusion of participants.

Participants and investigators were not informed about the allocation of participants. However, the commercially available products were provided in their normal packages with mandatory nutrition labelling information. Therefore, based on the used products, researchers could identify the allocation of participants. In order to optimise blinding, participants were neither informed that protein was the main nutrient of interest, nor were they informed which products the other group received. The researcher who performed the analyses was not blinded to the group allocation of participants, but the database was re-blinded before running the analyses.

### Treatment

Participants in the intervention group could choose from thirty-two home-delivered ready-made protein-rich hot meals and protein-rich dairy products (Table 1). The protein-rich meals contained at least 20% energy from protein and on average 30·5 (sd 5·8) g of protein. Participants in the control group could choose between thirty home-delivered standard (not classifying as protein-rich) ready-made hot meals and drinks (low-protein desserts or fruit juices; see Table 1). The protein content of these standard meals was on average 21·3 (sd 4·2) g per meal. During 4 weeks (28 consecutive days), participants received *ad libitum* drinks and dairy products (free choice). As a typical Dutch meal pattern is based on two bread meals and one hot meal, participants received one hot meal per d. All products provided were free of charge (total value of approximately 250 euro for the whole intervention period), chosen by participants themselves and delivered at home once a week. There was no possibility to order extra products between regular deliveries if participants ran out of products. Participants were asked to use one ready-made meal per d but the use of the other products was at their own choice. Besides the products provided in the trial, they were free to use any additional products they wanted. All products were commercially available and were provided by the Sligro Food Group and Friesland-Campina.

**Table 1.** Provided (dairy) products and ready-made meals during the ConsuMEER study

	Intervention group			Control group	
Products	Portion size	Protein per portion (g)	Products	Portion size	Protein per portion (g)
(Semi-skimmed) milk	150 ml	5·4	Juice (three flavours)	200 ml	0·6
Buttermilk	150 ml	5·4	Custard (three flavours)	150 ml	4·2
Drinking yoghurt			Butter	5 g	0·1
Greek style (three flavours)	150 ml	6·3			
Protein-rich (two flavours)	150 ml	7·8			
(Semi) skimmed yoghurt	150 ml	7·1			
Greek yoghurt (three flavours)	150 ml	11·6			
Cheese spreads	30 g	5·1			
Cheese (48+)	30 g	7·6			
Oatmeal porridge	150 ml	6·0			
Ready-made meals					
Protein-rich meals	500–550 g	30·5* (sd 5·8)	Standard meals	500–550 g	21·3* (sd 4·5)

* Average protein content.

### Measurements

Eleven trained students of the BSc programmes ‘Nutrition and Dietetics’, ‘Food Innovation’ and ‘Food Technology’ performed all measurements. Measurements were performed at three time points: baseline, T1 (2 weeks after the start of the intervention) and T2 (4 weeks after the start of the intervention). Students visited the participants at their homes in duos. During the trial, each participant was measured by the same set of students.

Baseline measurements were performed to obtain a participant's general health status. These measurements were only conducted at the start of the trial and included the following.

#### Mini Mental State Examination

The MMSE is a validated questionnaire containing nineteen items to assess cognitive performance^(19)^. A score below 24 (maximum score 30 points) indicates cognitive impairment^(20)^.

#### Seniors in the Community: Risk Evaluation for Eating and Nutrition, version II

Seniors in the Community: Risk Evaluation for Eating and Nutrition, version II (SCREEN II) is a validated questionnaire based on seventeen items to identify the risk for impaired nutritional status in community-living older adults. This questionnaire focuses on food habits that are associated with an impaired nutritional status. A score below 50 points (maximum score 64 points) indicates a high risk for impaired nutritional status^(21)^.

#### Short Nutritional Assessment Questionnaire 65+

The Short Nutritional Assessment Questionnaire 65+ (SNAQ65+) is a validated tool containing four items (involuntary weight loss, upper arm circumference, appetite and ability to walk stairs) to assess the risk of undernutrition in community-dwelling older adults. Persons are categorised into three groups: no risk of undernutrition, moderate risk of undernutrition and severe risk of undernutrition^(22)^.

#### Longitudinal Ageing Study Amsterdam Physical Activity Questionnaire

The Longitudinal Ageing Study Amsterdam Physical Activity Questionnaire (LAPAQ) is a validated tool containing eighteen items to assess physical activity of older people in the last 2 weeks. The LAPAQ provides information about physical activity in min/week. Activities can be categorised in low intensity (walking and low-intensity house-holding activities) and high intensity (bicycling, sports and high-intensity house-holding activities)^(23)^. Low activity was categorised as <150 min of high-intensity activities per week^(24)^.

#### Timed ‘Up & Go’

Timed ‘Up & Go’ is a validated tool to identify gait speed and mobility level in older people^(25)^. A time below 12 s is recognised as normal^(26)^. Participants who were not able to perform timed ‘Up & Go’ because of low mobility were categorised as >12 s.

#### Handgrip strength

Handgrip was measured by a Jamar dynamometer and expressed in kg. The Jamar dynamometer has been proven to be an accurate measurement tool for handgrip strength^(27)^. Sex-specific reference values of Dodds *et al*.^(28)^ were used for cut-off points; maximum handgrip strength (of both hands) below the 10th percentile (p10) was considered as low handgrip strength. Unperformed measurements because of medical conditions were categorised as low handgrip strength.

#### Weight

Participants' BW was measured twice at baseline, in clothes and without shoes, by using the scales of the participants. If the two measurements differed >0·1 kg, a third measurement was performed. The average of two measurements that were nearest was used.

#### Height

Participants’ height was measured twice at baseline, without shoes. If measurements differed >0·3 cm, a third measurement was performed. The average of two measurements that were nearest was used.

#### Co-morbidities

The number of co-morbidities was assessed by using a pre-specified list and was based on self-report. Co-morbidities were categorised as: high blood pressure, lung disease (asthma, chronic obstructive pulmonary disease, emphysema), stomach-liver-bowel disease, kidney or bladder disease, joint wear (arthrosis, rheumatism), osteoporosis, back disorders, diabetes, stroke or cerebral haemorrhage/infarction, heart attack, other severe heart problems (heart failure, angina pectoris), cancer and ‘other’.

### Dietary assessment

Participants were requested to fill out 3-d structured food diaries (‘gold standard’ for measuring food intake)^(29)^ at three time points: baseline, T1 and T2. Food diaries were delivered before the start of the study and participants were instructed on how to fill out the diaries. Additional information on filling out the diaries, including examples (e.g. weighing foods, describing fat content of dairy products) were provided in the first pages of the diaries. Diaries were pre-structured by three meal moments and three in-between moments. Food diaries were filled out by the participants themselves, 3 d before each measurement. Trained students of ‘Nutrition and dietetics’ checked the food diaries for completeness in collaboration with the participants during the home visits. Brands and types of products and quantity (g, volumes, sizes of cups/glasses, etc.) were asked if not registered by the participants.

The food diaries were digitised in Evry (Evry BV). This program is frequently used by dietitians in the Netherlands, to calculate food intake. Calculations within Evry are based on the Dutch Food Composition Table 2013^(30)^. Macronutrient (g) and total energy (kcal (kJ)) intakes of participants were calculated and reported as daily averages as well as protein intake per meal moment (three main meals and three in-between meals).

### Liking meals and compliance

A five-point scale was used to assess liking of meals, ranging from 1 (dislike a lot) to 5 (like a lot). Compliance of eating ready-made meals was based on recorded intake in the 3-d food diary.

### Ethics

The study was conducted according to the principles of the Declaration of Helsinki. The ethics committee of Radboud University Nijmegen Medical Centre evaluated the study and it was judged not to fall within the remit of the Medical Research Involving Human Subjects Act (WMO). All patients provided written informed consent before the start of the trial. Participants were allowed to retract from the study at any time point. If possible, reasons for retraction were asked.

### Data analysis

#### Sample size calculation

The primary outcome of this study was achieving a total protein intake of at least 1·2 g protein/kg BW per d. Secondary outcomes were daily protein intake (g), protein intake at breakfast, lunch and dinner (g) and achieving an intake of ≥25 g protein at every meal moment.

A sample size calculation was performed based on results of a previous study^(5)^, in which the average protein intake in community-dwelling older adults was shown to be 0·9 (sd 0·3) g/kg BW. As an intake of 1·2 (sd 0·3) g protein/kg BW per d is thought to be optimal^(7,8,31)^, an increase of 0·3 g protein/kg BW per d would be relevant. Based on a power of 90 % and an α of 5 %, a sample size of twenty-one participants was needed per group to detect this increase in protein intake. However, to compensate for loss to follow-up and to allow for analyses other than the primary outcome, a sample size of fifty participants per group was chosen.

Intention-to-treat analysis based on available cases was used for all data, meaning that all participants were analysed according to their group allocation. When participants withdrew from the study, unperformed measurements were recorded as missing data.

#### Confounding

Despite randomisation, potential confounders could possibly not be equally distributed over both groups. However, if confounders influence the protein intake of the participants, this effect is likely to be already present at the baseline measurement. Therefore, adjusting for protein intake at baseline measurement will correct for potential confounding. Furthermore, adjusting for protein intake at baseline will correct for regression to the mean. Therefore, ‘protein intake at baseline’ was added as a covariate in all analyses.

#### Analyses

Outcomes of continuous data were checked for normality using QQ-plots and box plots. Baseline characteristics were reported as means and standard deviations or medians and interquartile ranges (Q1–Q3) for continuous data and frequencies and percentages for categorical data. Linear mixed models were used to test for differences in continuous outcomes (protein intake in g and g protein/kg BW per d) between the intervention and control groups. A three-level structure was used to correct for clustering within the two measurements (T1 and T2) and within participants because of the clustered data of the 3-d food diary. Therefore, a random intercept was created at measurement and participant level. Allocated group and ‘protein intake at baseline’ were used as fixed-effects terms. Protein intake at baseline was added to the model at T1 and T2 as a fixed-effects term because of differences in protein intake at baseline between the intervention and control groups.

Within-group differences during the trial for protein intake in g and g protein/kg BW per d were tested by a multilevel analysis. Analyses were performed separately for the intervention and control groups with a random intercept at participants' level and ‘time point’ as a fixed-effects term.

Logistic regression was used to test for differences in the dichotomous outcomes of reaching the threshold of 1·2 g protein/kg BW per d and ≥25 g per meal moment. For each time point the average daily protein intake of each participant was calculated by aggregating the data of the 3-d food diaries and divided by the participant's BW. Thereafter, this variable was dichotomised in </≥1·2 g protein/kg BW per d. The same procedure was performed for the ≥25 g protein per meal moment; for each time point the average protein intake per meal moment was calculated and thereafter this variable was dichotomised into </≥25 g protein per meal.

The average meal acceptance during the trial was calculated per week and reported for the intervention and control groups separately. Differences between the intervention and control groups were tested with the unpaired *t* test.

All statistical analyses were performed using SPSS software 24 (IBM) and *P*<0·05 was considered significant.

## Results

**Fig. 1.**
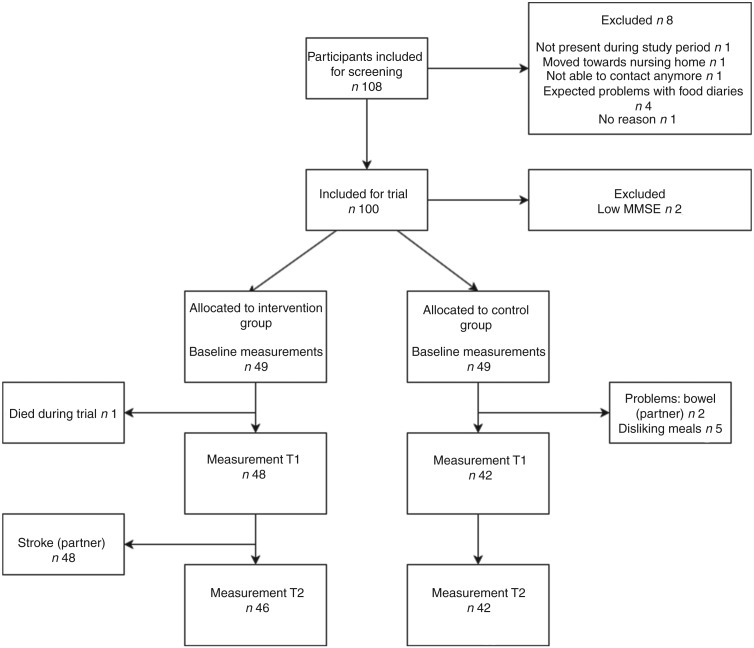
Flowchart of enrolment and dropout of participants during the ConsuMEER study. MMSE, Mini Mental State Examination; T1, 2 weeks after start of intervention; T2, 4 weeks after start of intervention.

A total of 100 participants were recruited to participate in the trial (Fig. 1). Two participants already used ready-made meals before the trial; both were allocated to the control group. After the baseline measurement two participants were found to have a low MMSE score and were therefore excluded. Of the remaining ninety-eight participants, forty-nine were allocated to the intervention group and forty-nine to the control group. During the trial, twelve participants (four in the intervention group and eight in the control group) were lost to follow-up due to various reasons (reasons are shown in Fig. 1).

### Baseline data

Participants’ characteristics are shown in Table 2; sixty (61 %) were female, mean age was 80·4 (sd 6·8) years and mean BMI was 27·9 (sd 5·0) kg/m^2^. Of the participants, seven were categorised with a low MMSE score (<24 points), but were included as their partners had an MMSE ≥24. Based on SCREEN II, forty-five participants had a risk of an impaired nutritional status (<50 points). In addition, twenty participants were at moderate or severe risk of undernutrition according to SNAQ65+, but none of them used oral nutritional supplements. As can be read from Table 2, more than 50 % of participants had a low activity level (by LAPAQ), almost 50 % had a prolonged timed Up & Go >12 s, and more than 15 % had a low grip strength. Most participants had one or more co-morbidities (91·8 %), had received middle (40·8 %) or high (30·6 %) education, had a higher income (54·1 %) and received home care or domestic help (66·3 %).

**Table 2.** Baseline characteristics of participants included in the ConsuMEER study (Frequencies and percentages; mean values and standard deviations; medians and interquartile ranges (Q1–Q3))

	Control (*n* 49)		Intervention (*n* 49)		Total (*n* 98)	
	*n*	%	*n*	%	*n*	%
Sex						
Male	20	40·8	18	36·7	38	38·8
Female	29	59·2	31	63·3	60	61·2
Age (years)						
Mean	80·6		80·2		80·4	
sd	6·7		7·0		6·8	
BMI (kg/m^2^)						
Mean	27·9		27·9		27·9	
sd	4·5		5·4		5·0	
Marital status						
Single	24	49·0	24	49·0	48	49·0
Couple	25	51·0	25	51·0	50	51·0
MMSE (points)						
Median	29		28		29	
Q1–Q3	27–30		26–30		26–30	
<24 points	1	2·0	6	12·2	7	7·1
SCREEN II (points)						
Mean	50·1		49·0		49·6	
sd	6·4		7·0		6·7	
<50 points	23	46·9	22	44·9	45	45·9
SNAQ65+						
No risk of undernutrition	39	79·6	39	79·6	78	79·6
Moderate risk undernutrition	7	14·3	3	6·1	10	10·2
Severe risk undernutrition	3	5·9	7	14·3	10	10·2
LAPAQ (min/d)						
Low-intensity activity						
Median	65·0		76·1		75·0	
Q1–Q3	45·0–120·0		38·9–120·0		40·0–120·0	
High-intensity activity						
Median	15·0		21·4		16·8	
Q1–Q3	2·1–33·9		4·3–42·9		2·1–42·9	
Low activity*	33	67·3	26	53·1	59	60·2
Timed ‘Up & Go’ (s)						
Median	10·3		10·1		10·3	
Q1–Q3	8·9–13·6		8·5–13·9		8·7–13·7	
>12 s†	22	44·9	17	34·7	39	39·8
Not performed	5	10·2	3	6·1	8	8·2
Handgrip strength (kg)						
Median	24·0		28·5		26·5	
Q1–Q3	20·8–31·5		20·0–34·9		20·1–32·1	
Low handgrip‡	8	16·3	7	14·3	15	15·3
Not performed	3	6·1	3	6·1	6	6·1
Co-morbidities (amount)						
Median	3		3		3	
Q1–Q3	1–4		1–4		1–4	
No co-morbidities	3	5·9	6	12·2	9	8·2
Education						
Low	7	14·3	16	32·7	23	23·5
Middle	25	51·0	15	30·6	40	40·8
High	16	32·7	14	28·6	30	30·6
Missing	1	2·0	4	8·2	5	5·1
Income§						
Low	17	34·7	28	57·1	45	45·9
High	32	65·3	21	42·9	53	54·1
Help at home||						
Home care or domestic help	31	63·3	34	69·4	65	66·3
No help	18	36·7	15	30·6	33	33·7

MMSE, Mini Mental State Examination; SCREEN II, Seniors in the Community: Risk evaluation for eating and nutrition, version II; SNAQ65+, Short Nutritional Assessment Questionnaire 65+; LAPAQ, Longitudinal Ageing Study Amsterdam Physical Activity Questionnaire.

* Below 150 min of high-intensity activities per week.

† >12 s or not performed because of low mobility.

‡ Below 10th percentile (p10) of Dodds *et al*.^(28)^ or not performed because of medical conditions.

§ Low income was defined as annual income <€28 500 for singles or <€35 000 for couples.

|| Combinations of help at home possible.

### Baseline nutritional intake

As shown in Table 3, protein intakes at breakfast and lunch were comparable between both groups and neither group reached the threshold of 25 g of protein. At dinner, for both groups average intake reached the threshold of 25 g of protein. At this meal moment, the intake of the intervention group was 38·7 (se 2·5) g and in the control group 33·7 (se 2·2) g. Daily protein intake was 1·09 (se 0·05) g protein/kg BW per d in the intervention group and 0·99 (se 0·05) g protein/kg BW per d in the control group.

**Table 3.** Protein intakes per meal moment and differences (g) between the intervention and control groups; energy intakes (Mean values with their standard errors; mean differences and 95% confidence intervals)

	Control		Intervention		Between-group difference†		Between-group difference adjusted for baseline protein intake†	
	Mean	se	Mean	se	Mean	95% CI	Mean	95% CI
Breakfast protein intake								
Baseline (*n* 49/49)	14·1	0·9	13·4	1·0	−0·7	−3·2, 1·7	–	–
During trial (T1 *n* 42/48) (T2 *n* 42/46)	14·8	0·8	15·4	0·7	0·6	−1·5, 2·6	1·4	−0·3, 3·1
Lunch protein intake								
Baseline (*n* 49/49)	15·9	1·2	18·2	1·1	2·3	−1·0, 5·5	–	–
During trial (T1 *n* 42/48) (T2 *n* 42/46)	15·3	1·0	18·4	1·0	3·2*	0·5, 5·9	2·0*	0·0, 4·0
Dinner protein intake								
Baseline (*n* 49/49)	33·7	2·2	38·7	2·5	5·1	−1·1, 11·3	–	–
During trial (T1 *n* 42/48) (T2 *n* 42/46)	24·5	0·9	34·8	0·9	10·3*	7·8, 12·9	9·6*	7·1, 12·1
Daily total protein intake								
Baseline (*n* 49/49)	73·9	3·3	80·4	4·1	6·5	−3·0, 16·0	–	–
g protein/kg BW per d	0·99	0·05	1·09	0·05	0·10	−0·04, 0·23	–	–
During trial (T1 *n* 42/48) (T2 *n* 42/46)	64·4	2·0	79·8	1·9	15·4*	10·0, 20·8	13·6*	9·0, 18·3
g protein/kg BW per d	0·87	0·03	1·12	0·05	0·24*	0·15, 0·33	0·23*	0·14, 0·31
Daily total energy intake								
Baseline (*n* 49/49)								
kcal	1871	76	2037	78	165	−51, 183	–	–
kJ	7828	318	8523	326	690	−213, 766	–	–
During trial (T1 *n* 42/48) (T2 *n* 42/46)								
kcal	1865	47	1795	45	−71	−199, 58	–	–
kJ	7803	197	7510	188	−297	−833, 243	–	–

T1, 2 weeks after start of intervention; T2, 4 weeks after start of intervention; BW, body weight.

* Significant difference (*P*<0·05).

† Based on multilevel analysis.

### Intervention effects

#### Effect of the ready-made meals and dairy products on protein intake

The total protein intake of participants in the intervention group was higher than that of the control group (mean difference: 13·6 (95% CI 9·0, 18·3) g). Despite a 0·23 (95% CI 0·14, 0·31) g protein/kg BW per d higher protein intake in the intervention group, neither the intervention nor the control group reached the average daily protein goal of 1·2 g protein/kg BW per d. At breakfast and lunch, differences in protein intake between groups remained small (<2 g protein difference) but were significant at lunch (mean difference 2·0 (95% CI 0·0, 4·0) g) (Table 3). Also, a significantly higher intake of protein (g) was seen at dinner for the intervention group compared with the control group (mean difference: 9·6 (95% CI 7·1, 12·1) g).

In the control group, protein intake decreased compared with baseline, both at dinner and total protein per d (*P* < 0·05). In the intervention group, a higher intake compared with baseline was seen at breakfast at both time points, while intake at dinner decreased at T1 but this was not seen at T2. No differences (*P* > 0·05) were seen in daily protein intake in the intervention group compared with baseline. Also, for all meal moments no differences (*P* > 0·05) were seen between T1 and T2 for either the intervention or control group.

#### Reaching threshold of 1·2 g protein/kg body weight per d or 25 g per meal moment

In Table 4, the percentages/proportions of participants reaching 1·2 g protein/kg BW per d and/or ≥25 g of protein per meal are shown. More participants reached an intake of 1·2 g protein/kg BW per d in the intervention group compared with the control group (T1 OR 4·85 (95% CI 1·59, 14·80); T2 OR 3·56 (95% CI 1·15, 11·14)). Despite the higher odds for the intervention group, only one-third of all participants reached the threshold of 1·2 g protein/kg BW per d in this group (T1: 34·8 %, T2: 32·6 %).

**Table 4.** Incidence and risk for reaching 25 g protein per meal moment or 1·2 g protein/kg body weight (BW) per d (Numbers and percentages; odds ratios and 95% confidence intervals)

	Control		Intervention		Effect size†		Effect size adjusted for baseline protein intake†	
	Number	%	Number	%	OR	95% CI	OR	95% CI
Daily 1·2 g protein/kg BW per d								
Baseline (*n* 49/49)	13	26·5	15	31·9	1·42	0·60, 3·45	–	–
T1 (*n* 42/48)	4	9·5	16	34·8	5·20*	1·73, 15·69	4·85*	1·59, 14·80
T2 (*n* 42/46)	5	11·9	14	32·6	3·57*	1·15, 1107	3·56*	1·15, 11·04
Breakfast ≥25 g protein/meal								
Baseline (*n* 49/49)	1	2	4	8·5	N.A.	–	N.A.	–
T1 (*n* 42/48)	2	4·8	4	8·7	N.A.	–	N.A.	–
T2 (*n* 42/46)	1	2·4	4	9·3	N.A.	–	N.A.	–
Lunch ≥25 g protein/meal								
Baseline (*n* 49/49)	6	12·2	7	14·9	1·25	0·39, 4·05	–	–
T1 (*n* 42/48)	5	11·9	11	23·9	2·33	0·73, 7·37	2·99	0·70, 12·86
T2 (*n* 42/46)	6	14·3	9	20·9	1·59	0·51, 4·94	1·41	0·43, 4·63
Dinner ≥25 g protein/meal								
Baseline (*n* 49/49)	34	69·4	42	89·4	3·71*	1·22, 11·22	–	–
T1 (*n* 42/48)	18	42·9	39	84·8	7·43*	2·70, 20·40	8·24*	2·73, 24·83
T2 (*n* 42/46)	17	40·5	38	88·4	11·18*	3·66, 34·17	11·99*	3·70, 38·83

T1, 2 weeks after start of intervention; T2, 4 weeks after start of intervention; N.A., not applicable due to a low incidence.

* Significant difference (*P*<0·05).

† Based on logistic regression.

The higher intake of protein in the intervention group was also reflected in the proportions of participants reaching the threshold of 25 g of protein per meal moment. At breakfast and lunch, no significant differences were seen between the intervention and control groups. At dinner, participants of the intervention group were more likely to reach an intake of 25 g protein compared with the control group (T1 OR 8·24 (95% CI 2·73, 24·83), T2 OR 11·99 (95% CI 3·70, 38·83)).

#### Protein intake derived from dairy products and ready-made meals

*Post hoc* analyses regarding protein intake from dairy products and ready-made meals are shown in Table 5. The use of ready-made meals resulted in an average daily intake of 25·8 (se 0·5) g of protein in the intervention group compared with 18·5 (se 0·5) g in the control the group; this difference was significant (difference 6·9 (95% CI 5·5, 8·3) g). Daily protein intake from dairy products was significantly higher in the intervention group compared with the control group at every meal moment. The largest difference was observed at dinner where the intervention group consumed 3·9 (95% CI 2·3, 5·6) g of protein (derived from dairy products) more than the control group. The total daily difference of protein derived from dairy products between the control and intervention groups was 9·6 (95% CI 6·2, 13·1) g.

**Table 5.** Protein intake (g) from dairy products and ready-made meals during the ConsuMEER study (Mean values with their standard errors; mean differences and 95% confidence intervals)

	Control		Intervention		Between-group difference†		Between-group difference adjusted for baseline protein intake†	
	Mean	se	Mean	se	Mean	95% CI	Mean	95% CI
Ready-made meals								
During trial (T1 *n* 42/48) (T2 *n* 42/46)	18·5	0·5	25·8	0·5	7·2*	5·8, 8·7	6·9*	5·5, 8·3
Breakfast dairy products								
Baseline (*n* 49/49)	4·3	0·5	4·4	0·5	0·1	−1·4, 1·5	–	–
During trial (T1 *n* 42/48) (T2 *n* 42/46)	4·7	0·6	6·6	0·6	1·9*	0·3, 3·6	2·2*	0·6, 3·8
Lunch dairy products								
Baseline (*n* 49/49)	5·2	0·6	6·9	0·6	1·7	−0·1, 3·5	–	–
During trial (T1 *n* 42/48) (T2 *n* 42/46)	4·7	0·6	7·7	0·6	3·0*	1·3, 4·8	2·5*	0·9, 4·1
Dinner dairy products								
Baseline (*n* 49/49)	3·4	0·5	4·9	0·5	1·5*	0·0, 3·0	–	–
During trial (T1 *n* 42/48) (T2 *n* 42/46)	3·5	0·6	7·7	0·6	4·2*	2·6, 5·8	3·9*	2·3, 5·6
Daily total dairy products								
Baseline (*n* 49/49)	16·0	1·2	19·5	1·3	3·5	−0·1, 7·0	–	–
During trial (T1 *n* 42/48) (T2 *n* 42/46)	15·8	1·3	26·1	1·3	10·3	6·7, 13·9	9·6	6·2, 13·1

T1, 2 weeks after start of intervention; T2, 4 weeks after start of intervention.

* Significant difference (*P*<0·05).

† Based on multilevel analysis.

#### Liking and compliance

Overall acceptance of meals was 3·6 (sd 0·5) in the control group and 3·6 (sd 0·6) in the intervention group based on a five-point scale (mean difference: 0·0 (95% CI −0·2, 0·3)).

Based on the 3-d food diaries, participants in the intervention group used ready-made meals on 88·9 % of the registered days at T1 and on 79·7 % of the days at T2. In the control group these numbers were 92·1 % at T1 and 84·1 % at T2.

## Discussion

The aim of the present study was to test the effectiveness of commercially available protein-rich ready-made meals and protein-rich dairy products in increasing protein intake, after switching from self-prepared meals towards home-delivered ready-made meals. In contrast to expectations, results show that switching from self-prepared meals towards standard ready-made meals turned out to be a risk for a decreasing protein intake; this effect was not seen in participants who used protein-rich ready-made meals and protein-rich dairy products. Both ready-made meals and dairy products contributed to this higher intake of the intervention group. Although participants receiving products high in protein consumed more protein than participants receiving products with lower protein content, their total protein intake did not increase compared with their pre-study protein intake and did not reach the goal of 1·2 g protein/kg BW per d. Moreover, not all participants of the intervention group reached the recommended intake of >25 g protein at dinner.

The characteristics of the participants included in the present ConsuMEER study showed only minor differences compared with the Dutch Longitudinal Ageing Study Amsterdam (LASA) cohort^(32)^ and participants of the Dutch National Food Consumption Survey Older Adults (DNFCSOA)^(6)^. Overall age of the participants in the ConsuMEER study (80·4 (sd 6·7) years) was slightly higher when compared with the DNFCSOA (mean age: male 76·6 (sd unknown) years, female 78·4 (sd unknown) years) and LASA cohort (77·3 (sd 6·7) years). BMI of participants in the ConsuMEER study was relatively high (male: 27·1 (sd 4·1) kg/m^2^; female 28·4 (sd 5·4) kg/m^2^) and comparable with the BMI reported in the DNFCSOA (27·1 (sd unknown) kg/m^2^) and BMI of female participants of the LASA cohort (27·9 (sd 4·8) kg/m^2^). However, the BMI was higher compared with that of male participants of the LASA cohort (25·9 (sd 3·4) kg/m^2^). In the ConsuMEER study, 21 % of all participants were at moderate or severe risk of undernutrition according to SNAQ65+, in LASA, this percentage was 18·3 % and in the DNFCSOA, this was 13·1 %. Protein intake of the participants of the ConsuMEER study at baseline was comparable with the protein intake of the DNFCSOA where on average 1·01 (sd unknown) g protein/kg BW per d was consumed. Thus, despite some minor differences in participants' characteristics with previous performed studies, the participants of the ConsuMEER study seem to be a good reflection of older adults in the Netherlands.

The ConsuMEER study is one of the first trials investigating the protein intake of community-dwelling older adults when changing from self-prepared meals towards commercially available home-delivered, ready-made meals. The protein intake in the control group decreased, while the protein intake in the intervention group remained stable. In addition, our results suggest that protein-rich ready-made meals and protein-rich dairy products are effective in reaching the threshold of ≥25 g protein at dinner but not at breakfast and lunch. Because of the low intake at breakfast and lunch, the threshold of 1·2 g protein/kg BW per d was not reached for most participants. These results are in line with the results of a recent study by Denissen *et al*.^(33)^ where ready-made meals containing as much as 40 g of protein per meal were not sufficient in reaching 1·2 g protein/kg BW per d either. Our results are not in line with earlier studies from the USA though. These studies showed that standard ready-made meals were associated with higher protein intake^(34)^. However, most of these studies were performed in participants receiving ready-made meals because of the ‘Older Americans Act’ (OAA). People who are dependent of meals from the OAA are generally poor and therefore they may not have had enough money to buy relatively expensive products like meat and dairy products^(15)^. In those studies, the provided ready-made meals may therefore not have replaced self-prepared meals but may have been used instead of skipping a meal. This could explain the higher protein intake after switching towards ready-made meals in these studies.

The observed decrease in protein intake at dinner of the control group after switching from self-cooked meals to regular ready-made meals was not seen in a comparable study of Ziylan *et al*.^(17)^. In this study, the protein intake of the control group at dinner remained unchanged. This could be explained by the higher protein content of the standard meals in the control group of the Ziylan *et al*.^(17)^ study compared with the ConsuMEER study (average protein content Ziylan *et al*.^(17)^ 27·9 (sd 3·4) *v.* 21·3 (sd 4·5) in our study). Both the results of Ziylan *et al*.^(17)^ and the results of the present study indicate that a good choice of protein-rich ready-made meals and dairy products is necessary to prevent older adults from a decreasing protein intake after switching from self-prepared meals towards ready-made meals. This is of great interest because in the upcoming years, a larger group of older adults will have to rely on ready-made meals due to the ageing society and government policy of living at home as long as possible. Protein-rich ready-made meals and protein-rich dairy products could help to maintain a healthy nutritional status in older adults. Switching towards ready-made meals also has a downside. Meal preparation is important as it could provide joy to older adults^(35)^ and makes them feel independent^(36)^. It is also important that they keep cooking themselves to stay active, as a loss in daily activities cannot always be reversed^(37,38)^ and daily activities have a beneficial effect on cognitive functioning^(39)^. Therefore, older adults should try to keep preparing their own meals as long as possible.

During the trial a (borderline) significant difference in protein intake derived from dairy products between the control and intervention groups was seen at breakfast and lunch. However, this difference was relatively small (<3 g) and therefore protein intake in the intervention group still did not reach the threshold of ≥ 25 g of protein per meal moment. These results are in contrast to the study by van Til *et al*.^(18)^ where intake during breakfast and lunch was over 25 g. However, in this trial both protein-enriched dairy products (8 g protein per 100 ml; products not commercially available) and protein-enriched bread were used. Regular dairy products may be insufficient in increasing intake towards the recommended levels because of the large gap between regular intake at breakfast and lunch and the recommendation of eating ≥25 g of protein. Because an intervention based on protein-enriched bread alone was also not sufficient in reaching the threshold of ≥25 g of protein per bread meal^(17)^, future interventions should focus on all food products within a meal.

One of the reasons for the low increase in protein intake at breakfast and lunch could be unawareness about the importance of protein among older adults. Before and during the trial, no information was given about protein being the target nutrient of the study or about healthy eating in general. Older adults are interested in eating healthy to stay healthy but on average their knowledge is low^(40,41)^. Healthy eating is mainly described as eating a varied diet and eating a sufficient amount of fruit and vegetables^(40)^. The importance of eating enough protein and the distribution of protein intake is generally unknown in older adults^(41,42)^. Because of the free choice in using the provided dairy products and the supposed lack of knowledge about the advantages of consuming these products, it is plausible that some participants did not use the provided products at all. Providing protein-rich dairy products, combined with dietary advice about the importance of protein, is thought to make an intervention based on protein-rich dairy products more successful.

Some limitations need to be discussed to place the results of the present study in perspective. First, the duration of the intervention was relatively short (28 d). Therefore, no information is available on whether the protein intake will be maintained over a longer time period. It is possible that eating ready-made meals for a longer period could result in less appreciation for these meals, which could result in a lower intake. However, in the recent study of Denissen *et al*.^(33)^, where ready-made meals were used for a period of 3 months, overall appreciation of the meals remained high, also over a longer period of time. Another limitation is the use of one supplier for the ready-made meals; different brands of ready-made meals will differ in sensory aspects and macronutrient composition. Finally, the effect of the intervention on physical parameters was not measured. However, no changes were expected considering the intervention lasted only 4 weeks^(43)^. It can be argued that 1·2 g protein/kg BW per d may be a too high a goal for community-dwelling older adults. Based on expert opinion, a protein intake of 1·0–1·2 g protein/kg BW per d for healthy older adults and 1·2–1·5 g protein/kg BW per d for older adults with acute or chronic illness, and even up to 2·0 g protein/kg BW per d when severe illness is present is advised^(7)^. Based on a median of three co-morbidities in our study population, we considered our participants not fully healthy; therefore, we decided that a cut-off of 1·2 g protein/kg BW per d would be appropriate. Finally, a true control group is missing as both groups received an intervention. As both groups received an intervention, an option to overcome this issue could have been the use of a cross-over design in which group 1 starts with the intervention, followed by a control period, and group 2 starts with a control period, and then the intervention.

One of the strengths of this study is the high rate of follow-up (drop-out rate only 12 %). This could indicate that the intervention was easy to implement in daily life of participants and was well tolerated. This is of great importance because switching towards ready-made meals is likely to be a lasting change for older adults who are no longer capable of cooking their own meals. Another strength of the study is that it is similar to daily practice; the products that were used are commercially available, well known and fit in the daily lifestyle of Dutch older adults. Previous studies showed that older adults preferred the use of products they are familiar with in order to increase protein intake^(41,44)^. Also, no information about healthy eating was given and participants were free to make their own choices. Therefore, the results of the control group give valuable information on how protein intake can decrease when older adults change from self-prepared to ready-made meals without information about healthy eating.

Because of the relatively high protein intake at dinner and the importance of distribution of protein intake over meals, future interventions should focus on protein intake at breakfast and lunch. At these meal moments, the highest increase in protein intake could be achieved. Regular dairy products have the right nutritional composition to increase protein intake, are low in price, are easily accessible and are common in Dutch eating habits. Further qualitative research is needed on how older adults can be motivated to increase their protein intake at breakfast and lunch, as they are known to struggle to change their regular eating habits^(45,46)^.

### Conclusion

Switching from a regular diet to ready-made meals carries the risk of a decreasing protein intake if meals are not selected for high protein content. Protein-rich ready-made meals and protein-rich dairy products could prevent older adults from a decrease in protein intake but the combination of products provided in the present trial was not effective in increasing protein intake towards 1·2 g protein/kg BW per d. More research is needed concerning whether additional advice about protein intake could make an intervention based on regular protein-rich products more effective.
